# Global Practical Tracking by Output Feedback for Nonlinear Systems with Unknown Growth Rate and Time Delay

**DOI:** 10.1155/2014/713081

**Published:** 2014-09-03

**Authors:** Xuehua Yan, Xinmin Song

**Affiliations:** ^1^School of Electrical Engineering, University of Jinan, Jinan, Shandong 250022, China; ^2^School of Information Science and Engineering, Shandong Normal University, Jinan, Shandong 250014, China

## Abstract

This paper is the further investigation of work of Yan and Liu, 2011, and considers the global practical tracking problem by output feedback for a class of uncertain nonlinear systems with not only unmeasured states dependent growth but also time-varying time delay. Compared with the closely related works, the remarkableness of the paper is that the time-varying time delay and unmeasurable states are permitted in the system nonlinear growth. Motivated by the related tracking results and flexibly using the ideas and techniques of universal control and dead zone, an adaptive output-feedback tracking controller is explicitly designed with the help of a new Lyapunov-Krasovskii functional, to make the tracking error prescribed arbitrarily small after a finite time while keeping all the closed-loop signals bounded. A numerical example demonstrates the effectiveness of the results.

## 1. Introduction

As well known that the presence of time delay has a significant effect on system performance, it often causes deterioration of control system performance and may induce instability, oscillation, and poor performance in a large number of important physical, industrial, and engineering problems involving [1] networked control systems, information, or energy transportation. Therefore, the study of time delay systems has important practical significance and has received much attention in recent years [1–18]. Generally speaking, control design methods for time delay systems can be classified into two categories: delay-dependent [2–4, 11] and delay-independent [5–10].

From the survey on the problems of delayed systems in [19], there still have been many research issues coming up in the control problems of delayed systems. In this paper, we are concerned with the practical tracking for a more general class of uncertain nonlinear systems in the following form. (The following notations will be used throughout this paper. **R** denotes the set of all real numbers. **R** ^+^ denotes the set of all nonnegative real numbers. **R** ^*n*^ denotes the real *n*-dimensional space. For a given vector or matrix *X*, *X* ^*T*^ denotes its transpose; for any *x* ∈ **R** ^*n*^, ||*x*||_1_ denotes the 1-norm; that is, ||*x*||_1_ = |*x*_1_| + ⋯+|*x*_*n*_|; ||*x*|| denotes the Euclidean (or 2-) norm of vector *x*, and for the matrix *P*, we use ||*P*|| to denote its norm induced by the 2-norm of the corresponding vector; for any *x* ∈ **R** ^*n*^, there always holds ${||x||}_{1}\leq \sqrt{n}||x||$ .) Consider

(1) η˙i=ηi+1+ψi(t,η)+φi(t−d(t),η(t−d(t))),i=1,…,n−1,η˙n=u+ψn(t,η)+φn(t−d(t),η(t−d(t))),y=η1−yr,

where *η* = [*η*_1_,…, *η*_*n*_]^*T*^ ∈ **R** ^*n*^ is the system state vector with the initial value *η*_0_ = *η*(0);  *u* ∈ **R**,  *y* ∈ **R**, and *t* ↦ *y*_*r*_(*t*),  *t* ∈ **R** ^+^, are the control input, system output, and reference signal, respectively; *d*(*t*) : *R* → [0, *d*] is the time-varying time delay satisfying $\dot{d}(t)\leq \gamma <1$ for a known constant *γ*; and *ψ*_*i*_ : **R** ^+^ × **R** ^*n*^ → **R**, *φ*_*i*_ : **R** ^+^ × **R** ^*n*^ → **R**, *i* = 1,…, *n*, are unknown functions but continuous in the first argument and locally Lipschitz in the second one. In what follows, suppose only the system output is measurable.

The objective of the paper is to design an adaptive controller such that the resulting closed-loop system is well-defined and globally bounded on **R** ^+^, and furthermore, for any prescribed tracking precision *l* > 0 and every initial condition, there is a finite time *T*_*λ*_ > 0 such that sup⁡_*t*≥*T*_*λ*__ | *y*(*t*)| = sup⁡_*t*≥*T*_*λ*__ | *η*_1_(*t*) − *y*_*r*_(*t*)|≤*l* (as described in [20]). To make this possible, the following assumptions are imposed on system (1) and reference signal *y*_*r*_.

**Assumption 1 .**

There exists an unknown constant *θ*_1_ ≥ 0 such that

(2) $$\begin{array}{c} \left|\psi_{i}\left(t,\eta \right)\right|\leq \theta_{1}\left(\left|\eta_{1}\right|+\cdots +\left|\eta_{i}\right|\right)+\theta_{1},\;i=1,\ldots ,n. \end{array}$$

**Assumption 2 .**

There exists an unknown constant *θ*_2_ ≥ 0 such that

(3) |φi(t−d(t),η(t−d(t)))| ≤θ2(|η1(t−d(t))|+⋯+|ηi(t−d(t))|)+θ2,i=1,…,n.

**Assumption 3 .**

The reference signal *y*_*r*_ is continuously differentiable, and moreover, there is an unknown constant *M* ≥ 0 such that

(4) $$\begin{array}{c} \underset{t\geq 0}{\sup}\left(\left|y_{r}\left(t\right)\right|+\left|{\dot{y}}_{r}\left(t\right)\right|\right)\leq M. \end{array}$$

From Assumptions 1–3, it can be seen that the system investigated is substantially different from those of closely related tracking work [4, 10, 20] since the system considered in this paper contains not only the time delay term but also the unmeasured state dependent growth. In fact, [4, 10] consider the state feedback tracking problem, and in both of those papers, the assumption on reference signal is stronger than Assumption 3 in the paper. Although [20] studies global practical tracking problem by output feedback, it does not include the time delay.

## 2. Global Practical Tracking Control via Output Feedback

In the section, we design an adaptive output-feedback tracking controller for system (1) satisfying Assumptions 1–3 and prove that, with unknown time-varying time delay *d*(*t*) and unknown growth rate *θ* in Assumption 1 and without knowing the bound of the reference signal *y*_*r*_ and its derivation ${\dot{y}}_{r}$ in Assumption 3, the global practical tracking for the systems (1) can also be achieved.

First, with the help of the coordinates transformation *x*_1_ = *y*, *x*_*i*_ = *η*_*i*_, *i* = 2,…, *n*, system (1) becomes

(5) x˙1=x2+ϕ1(t,x,yr,y˙r) +φ1(t−d(t),x(t−d(t)),yr(t−d(t))),x˙i=xi+1+ϕi(t,x,yr) +φi(t−d(t),x(t−d(t)),yr(t−d(t))),i=2,…,n−1,x˙n=u+ϕn(t,x,yr) +φn(t−d(t),x(t−d(t)),yr(t−d(t))),

where $\phi_{1}(\cdot )=\psi_{1}(t,x_{1}+y_{r},x_{2},\ldots ,x_{n})-{\dot{y}}_{r}$ , *ϕ*_*i*_(·) = *ψ*_*i*_(*t*, *x*_1_ + *y*_*r*_, *x*_2_,…, *x*_*n*_), *i* = 2,…, *n*.

By Assumptions 1 and 2, for *i* = 1,…, *n*, it is easy to get the following inequalities:

(6) $$\begin{array}{c} \left|\phi_{i}\right|\leq \theta_{1}\left(\left|x_{1}\right|+\left|x_{2}\right|+\cdots +\left|x_{i}\right|\right)+\theta_{3}, \end{array}$$

(7) |φi(t−d(t),x(t−d(t)),yr(t−d(t)))| ≤θ2(|x1(t−d(t))|+|x2(t−d(t))|    +⋯+|xi(t−d(t))|)+θ4,

where *θ*_3_ = *θ*_1_(*M* + 1) + *M* > 0 and *θ*_4_ = *θ*_2_(*M* + 1) > 0 are unknown constants.

Then, motivated by [20], for any pregiven *l* > 0, we still construct the following adaptive tracking controller for system (5):


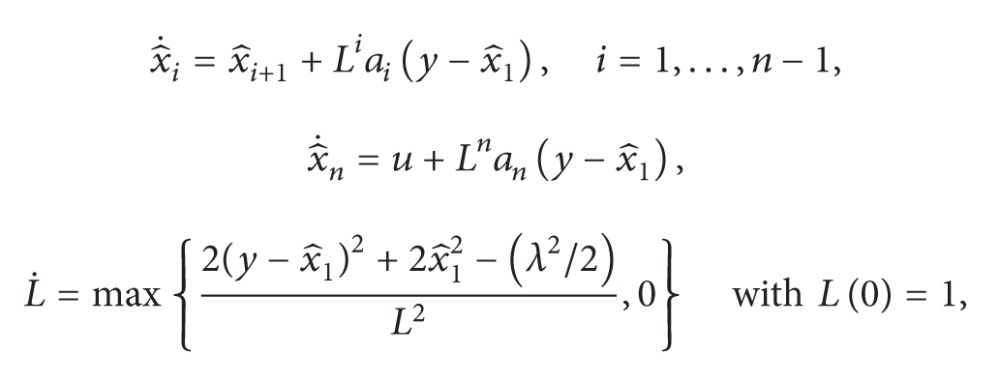

(8)



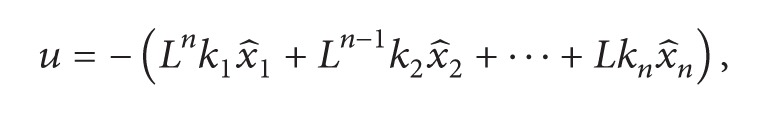

(9)

where *a*_*i*_ > 0 and *k*_*i*_ > 0, *i* = 1,…, *n*, are design parameters to be determined.

Similar to [20], before proving the claims of the theorem below, we first provide three fundamental propositions. The proof of Proposition 4 can be referred to Proposition 1 of [20]. The last two propositions are rigorously proven in Appendices A and B. Besides, by Proposition 4, it is not difficult to verify that the right-hand side of the resulting closed-loop system is continuous and locally Lipschitz in (*t*, *x*, *L*) in an open neighborhood of the initial condition, and hence the closed-loop system has a unique solution on a small interval [0, *t*_*s*_) (see Theorem 3.1, page 18 of [21]). Let [0, *t*_*f*_) be its maximal interval on which a unique solution exists, where 0 < *t*_*f*_ < +*∞* (see Theorem 2.1, page 17 of [21]).

**Proposition 4 .**

The gain *L* determined by (8) is monotone nondecreasing on its existence interval, and its dynamics are locally Lipschitz in $\left(y,{\hat{x}}_{1},L\right)$ .

**Proposition 5 .**

Define $e_{i}=x_{i}-{\hat{x}}_{i}$ , *ε*_*i*_ = *e*_*i*_/*L* ^*i*^, and $z_{i}={\hat{x}}_{i}/L^{i}$ , *i* = 1,…, *n*, and denote *ε* = [*ε*_1_,…, *ε*_*n*_]^*T*^, *z* = [*z*_1_,…, *z*_*n*_]^*T*^. Then, by choosing the suitable Lyapunov-Krasovskii functional *V*(*t*, *ε*, *z*, *L*), there holds on [0, *t*_*f*_)

(10) $$\begin{array}{c} \dot{V}\leq -\left(L-\Theta \right)\left({||\varepsilon ||}^{2}+{||z||}^{2}\right)+\frac{\Theta }{L^{2}}, \end{array}$$

where Θ is a positive constant.

**Proposition 6 .**

For the resulting closed-loop system, if *L* is bounded on [0, *t*_*f*_), then *z* and *ε* are bounded on [0, *t*_*f*_) as well.

**Remark 7 .**

Definitions of *ε*, *z* in Proposition 5 inspired by [20] are given the same as those in [20]. In fact, such definitions make it possible to offset the time delay term induced by the nonlinear time delay term *φ*_*i*_(·) by skilly choosing a Lyapunov-Krasovskii functional and thereby still can obtain the similar result (inequality (10)), which plays a key role in the proof of the theorem below. In addition, since Lyapunov-Krasovskii functional method can provide less conservative and delay-independent results than Razumikhin theorem approach, we use Lyapunov-Krasovskii functional method to design the controller of system (1) in this paper.

Now, we are in a position to state the following theorem, to summarize the main results of the paper. For the proof of the theorem, the reader is referred to Theorem 1 in [20].

**Theorem 8 .**

Under Assumptions 1–3, the global practical output-feedback tracking problem of system (1) can be solved by the dynamic output-feedback controller of forms (8) and (9).

## 3. An Illustrative Example

This section gives a numerical example to illustrate the effectiveness of Theorem 8.

**Example 1 .**

Consider the following uncertain nonlinear system:

(11) η˙1=η2+θ2η1(t−d(t)),η˙2=u−θ1sign⁡(η2)(1+|η2|),y=η1−yr,

where *d*(*t*) = (1/2)(1 + sin(*t*)) and sign⁡(·) denotes the signal function; that is, sign⁡(*x*) = 1( = −1) when *x* > 0(<0) and sign⁡(*x*) = 0 when *x* = 0; suppose *θ* = 0.5, *y*_*r*_ = sin(*t*).

**Figure 1:**
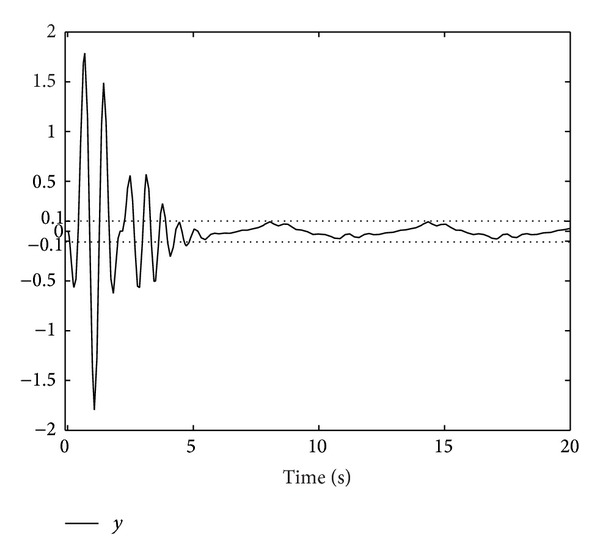
The trajectory of the tracking error *y*.

**Figure 2:**
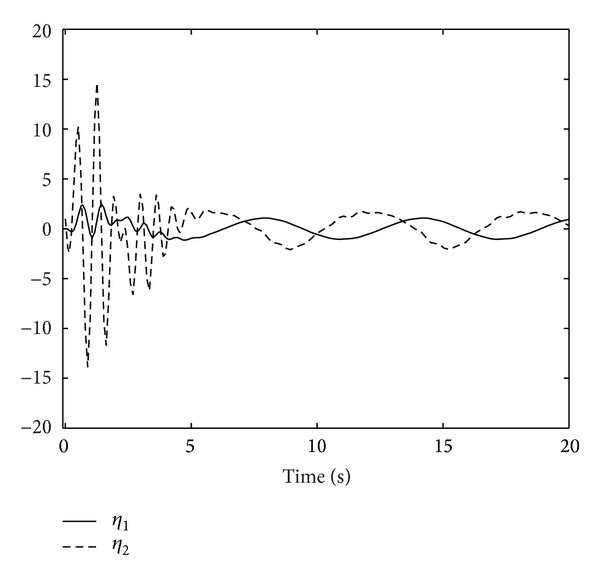
The trajectories of the system states *η*_1_ and *η*_2_.

**Figure 3:**
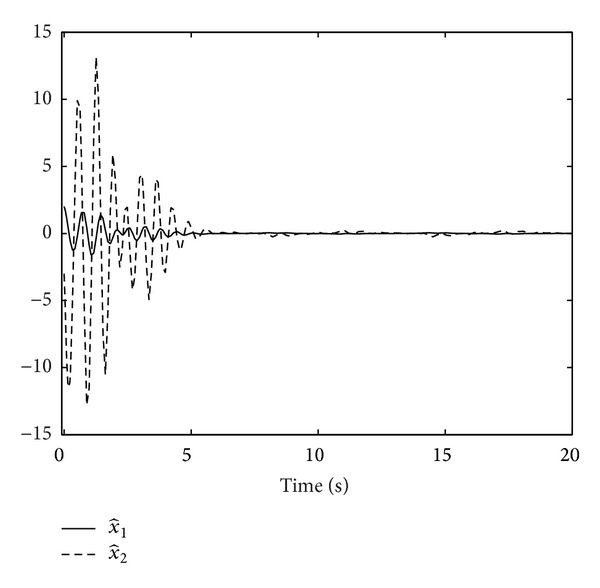
The trajectories of the observer states ${\hat{x}}_{1}$ and ${\hat{x}}_{2}$ .

**Figure 4:**
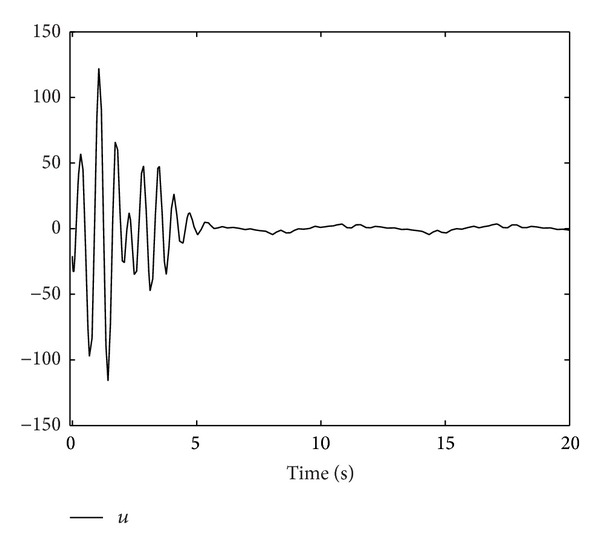
The trajectory of *u*.

**Figure 5:**
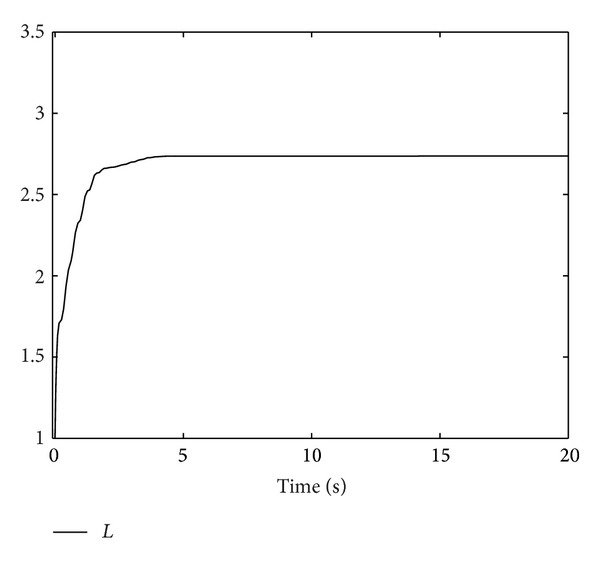
The trajectory of high-gain *L*.

Then a direct application of our proposed methodology yields a suitable adaptive output-feedback controller. Choose design parameters *a*_1_ = 1, *a*_2_ = 10, *k*_1_ = 12, and  *k*_2_ = 1. Let the tracking accuracy be *λ* = 0.1, and let initial conditions be *η*_1_(0) = 0, *η*_2_(0) = 1, ${\hat{x}}_{1}(0)=2$ , and ${\hat{x}}_{2}(0)=-3$ ; we obtain Figures 1, 2, 3, 4, and 5 by numerical simulation. From these figures, all the signals in the closed-loop system are bounded. From Figure 1, it can be seen that although the system contains time-varying time delay, after about seven seconds, the tracking error satisfies |*y*| = |*η*_1_ − *y*_*r*_| ≤ 0.1, which means that the prescribed tracking performance is achieved.

## 4. Conclusions

In this paper we extend the result in [20] to solve the global practical tracking problem for a class of nonlinear time delay systems by output feedback. Unlike most of the existing results, we allow the existence of unmeasurable states and time-varying time delay in the nonlinear growth. A stability analysis has been established based on the new Lyapunov-Krasovskii functional. The proposed controller independent of the derivative of time delay can make the tracking error arbitrarily small. Our future research is to extend the proposed framework for more general uncertain nonlinear systems, such as the systems with unknown control coefficients. Since the adopted controller in this paper is delay-independent, another topic of future work is to explore new delay-dependent, less conservative control design method.

## Appendices

Appendices A and B provide the rigorous proofs of fundamental Propositions 5 and 6, which are collected here for the sake of compactness.

### A. The Proof of Proposition 5

With the aid of the definitions of *e*_*i*_'s, *ε*_*i*_'s, and *z*_*i*_'s and by (5) and (8), one has

(A.1)
$$\begin{array}{c} {\dot{e}}_{i}=e_{i+1}-L^{i}a_{i}e_{1}+\phi_{i}\left(\cdot \right)+\varphi_{i}\left(\cdot \right),\;i=1,\ldots ,n-1,\\ {\dot{e}}_{n}=-L^{n}a_{n}e_{1}+\phi_{n}\left(\cdot \right)+\varphi_{n}\left(\cdot \right), \end{array}$$

(A.2)
ε˙=LAε+Φ(t,x,yr,y˙r,L) +Ψ(t−d(t),x(t−d(t)),yr(t−d(t)))−L˙LDε,z˙=LBz+Laε1−L˙LDz,

where *x* = [*x*_1_,…, *x*_*n*_]^*T*^, *a* = [*a*_1_,…, *a*_*n*_]^*T*^, and *D* = diag⁡{1,2,…, *n*},

(A.3)
Φ=[1Lϕ1(·),1L2ϕ2(·),…,1Lnϕn(·)]T,Ψ=[1Lφ1(·),1L2φ2(·),…,1Lnφn(·)]T,A=[−a11⋯0⋮⋮⋱⋮−an−10⋯1−an0⋯0]∈Rn×n,B=[01⋯0⋮⋮⋱⋮00⋯1−k1−k2⋯−kn]∈Rn×n.

In terms of Lemma 1 in [22], suitable constants *a*_*i*_'s and *k*_*i*_'s can be chosen such that the matrices *A*, *B* are Hurwitz ones and there exist *P* = *P* ^*T*^ > 0 and *Q* = *Q* ^*T*^ > 0 satisfying

(A.4)
$$\begin{array}{c} A^{T}P+PA\leq -I,\;\;DP+PD\geq 0,\\ B^{T}Q+QB\leq -2I,\;\;DQ+QD\geq 0. \end{array}$$

Keeping this in mind, choose the Lyapunov-Krasovskii functional *V* : **R** ^*n*^ × **R** ^*n*^ × **R** ^+^ → **R** ^+^

(A.5)
$$\begin{array}{c} V\left(t,\varepsilon ,z,L\right)=mV_{1}\left(\varepsilon \right)+V_{2}\left(z\right)+V_{3}\left(t,L\right) \end{array}$$

for system (A.2), where *m* = ||*Qa*||^2^ + 1 and *V*_*i*_, *i* = 1,2, 3, defined by

(A.6)
V1(ε)=εTPε,V2(z)=zTQz,V3(t,L)=m||P||1−γ∑i=1n∫t−d(t)t1L2i(s)φi2(s,x(s),yr(s))ds.

Then, on the interval [0, *t*_*f*_), along the solutions of (A.2), the derivative of *V* satisfies

(A.7)
V˙=−Lm||ε||2−mL˙LεT(DP+PD)ε−2L||z||2 −L˙LzT(DQ+QD)z+2mεTPΦ+2mεTPΨ +2Lε1zTQa+m||P||1−γ ×∑i=1n(1L2iφi2−1L2i(t−d(t))     ·φi2(t−d(t),x(t−d(t)),yr(t−d(t)))     ×(1−d˙(t))).

By supposition, *d*(*t*) ∈ [0, *d*], $\dot{d}\left(t\right)\leq \gamma <1$ , and noticing that *L*(*t*) is nondecreasing with *L* ≥ 1, the following inequality holds:

(A.8)
−m||P||1−γ1L2i(t−d(t))φi2 ×(t−d(t),x(t−d(t)),yr(t−d(t)))(1−d˙(t))≤−m||P||1L2i(t)φi2 ×(t−d(t),x(t−d(t)),yr(t−d(t))), ∀t∈[0,tf).

This, together with (A.4) and the fact that $\dot{L}\geq 0$ , *L* ≥ 1 on [0, *t*_*f*_), leads to

(A.9)
V˙≤−Lm||ε||2−2L||z||2+2mεTPΦ +2mεTPΨ+2Lε1zTQa+m||P||1−γ∑i=1n1L2iφi2 −m||P||∑i=1n1L2iφi2(t,x(t−d(t)),yr(t−d(t))).

By the method of completing square, we have

(A.10)
|2mεTPΦ|≤6θmn||P||(||ε||2+||z||2)+4θ12m||P||θL2,m||P||1−γ∑i=1n1L2iφi2≤4θ22mn2||P||1−γ(||ε||2+||z||2)+2θ42mn||P||(1−γ)L2,|2mεTPΨ|≤m||P||(||ε||2+||z||2) +m||P||∑i=1n1L2iφi2       ×(t−d(t),x(t−d(t)),         yr(t−d(t))),2Lε1zTQa≤L||z||2+L||Qa||2||ε||2.

Thus, setting Θ = max⁡{(6*θn* + 1)*m*||*P*|| + (4*θ*_2_^2^ *mn* ^2^||*P*||/(1 − *γ*)), (4*θ*_1_^2^ *m*||*P*||/*θ*)+(2*θ*_4_^2^ *mn*||*P*||/(1 − *γ*))}, from these inequalities and (A.9), it follows that (10) holds on [0, *t*_*f*_), and this completes the proof of Proposition 5.

### B. The Proof of Proposition 6

Notice that *L*(*t*_*f*_) = sup⁡_0≤*t*<*t*_*f*__ *L*(*t*) since *L* is monotone nondecreasing, continuous, and bounded on [0, *t*_*f*_). Along the same line as A2 of [20], the boundedness of *z* on [0, *t*_*f*_) is easily obtained. Then, let us show that *ε* is bounded on [0, *t*_*f*_). To this end, we introduce the change of coordinates *ξ*_*i*_ = *e*_*i*_/(*L**)^*i*^,  *i* = 1,…, *n*, where *L** is a constant satisfying

(B.1)
$$\begin{array}{c} L^{*}\geq \max\left\{L\left(t_{f}\right),\left(6\theta_{1}n+1\right)||P||+\frac{4\theta_{2}^{2}n^{2}||P||}{1-\gamma }+3\right\}. \end{array}$$

Then, the error dynamics (A.1) is transformed into

(B.2)
ξ˙=L∗Aξ+L∗aξ1−LΓaξ1+Φ∗(t,x,yr,y˙r) +Ψ∗(t−d(t),x(t−d(t)),yr(t−d(t))),

where Γ = diag⁡{1, *L*/*L**,…, (*L*/*L**)^*n*−1^}, Φ* = [*ϕ*_1_/*L**,*ϕ*_2_/(*L**)^2^,…,*ϕ*_*n*_/(*L**)^*n*^]^*T*^, and Ψ* = [*φ*_1_/*L**,*φ*_2_/(*L**)^2^,…,*φ*_*n*_/(*L**)^*n*^]^*T*^.

For system (B.2), we choose

(B.3)
V4(t,ξ) =ξTPξ+||P||1−γ∑i=1n∫t−d(t)t1(L∗)2iφi2(s,x(s),yr(s))ds.

Similar to (A.8), one readily gets

(B.4)
−||P||γ−11(L∗)2iφi2(t−d(t),x(t−d(t)),yr(t−d(t)))  ×(1−d˙(t)) ≤−||P||1(L∗)2iφi2(t−d(t),x(t−d(t)),yr(t−d(t))),                        ∀t∈[0,tf),

and then, on [0, *t*_*f*_), differentiating function *V*_4_ yields that

(B.5)
V˙4≤−L∗||ξ||2+2ξ1L∗aTPξ−2ξ1LaTΓPξ +2Φ∗TPξ+2Ψ∗TPξ+||P||1−γ∑i=1n1(L∗)2iφi2 −||P||∑i=1n1(L∗)2iφi2     ×(t−d(t),x(t−d(t)),yr(t−d(t))).

With the definitions of *ε*_*i*_ and *ξ*_*i*_, using (6), (7), and (B.1), by the method of completing the square, we obtain

(B.6)
|2ξ1L∗aTPξ|≤L∗2||aTP||2ξ12+||ξ||2,|2ξ1LaTΓPξ|≤L2||aTΓP||2ξ12+||ξ||2,|2Ψ∗TPξ|≤||P||||ξ||2+||P|| ×∑i=1n1(L∗)2iφi2(t−d(t),x(t−d(t)),         yr(t−d(t))),|2Φ∗TPξ|≤6θ1n||P||(||ξ||2+||z||2)+4||P||θ32θ1,||P||1−γ∑i=1n1L2iφi2≤4θ22n2||P||1−γ(||ξ||2+||z||2)+2θ42n||P||1−γ,

which, together with (B.1), means that, on [0, *t*_*f*_),

(B.7)
$$\begin{array}{c} {\dot{V}}_{4}\leq -\frac{1}{\lambda_{\max}\left(P\right)}V_{4}+\theta_{5}\dot{L}+\theta_{5}{\left(\underset{0\leq t<t_{f}}{\sup}||z\left(t\right)||\right)}^{2}+\theta_{5}\lambda^{2}+\theta_{5}, \end{array}$$

where *θ*_5_ > 0 is a constant. From this, one easily gets that *ξ* is bounded on [0, *t*_*f*_). Moreover, by the definitions of *ε*_*i*_ and *ξ*_*i*_ and the boundedness of *z* (just proved), *ε* is bounded on [0, *t*_*f*_).
